# Validity and reliability of Persian version of Low Physical Activity Questionnaire (LoPAQ)

**DOI:** 10.1186/s12882-024-03615-w

**Published:** 2024-05-22

**Authors:** Mohammad Ali Tabibi, Rahele Samouei, Nasrin Salimian, Shahrzad Shahidi, Abdolamir Atapour, Farzad Nazemi, Mahsa Ghenaat, Saghar Nikbakht, Mahboobeh Hoseini Sarbazi, Mahsa Soleymany, Zahra Roshanaeian, Behnaz Khajeheian, Zahra Khaki, Ali Sadeghi Sokani, Reyhane Ebrahimi, Saghar Ahmadi

**Affiliations:** 1 Department of Exercise Physiology, Pardis Specialized Wellness Institute, Isfahan, Iran; 2 Social Determinants of Health Research Center, Isfahan University of Medical Sciences, Isfahan, Iran; 3 Department of Research and Development, Pardis Specialized Wellness Institute, Isfahan, Iran; 4 Isfahan Kidney Diseases Research Center, Internal Medicine Department, Khorshid Hospital, Isfahan University of Medical Sciences, Isfahan, Iran; 5 Department of Kinesiology, Pardis Specialized Wellness Institute, Isfahan, Iran; 6 Department of Motor Behavior, Pardis Specialized Wellness Institute, Isfahan, Iran; 7 Department of Sport Nutrition, Pardis Specialized Wellness Institute, Isfahan, Iran; 8 Department of Health and Palliative Care, Pardis Specialized Wellness Institute, Isfahan, Iran

**Keywords:** Hemodialysis, Inactivity, Physical activity questionnaire, Validity, Reliability

## Abstract

**Background:**

The Low Physical Activity Questionnaire (LoPAQ) was specifically developed to measure the low activity level observed in extremely inactive hemodialysis (HD) patients. This study aims to evaluate reliability and validity of Persian version of the LoPAQ.

**Methods:**

This study was a cross sectional study, conducted in three HD centers in Iran. The LoPAQ was translated into Persian. After cultural adaptions, it was filled out by 120 HD patiens. Convergent validity, was evaluated by calculating the correlations among the Persian version of the LoPAQ and Persian version of the Community Healthy Adults Model Program for Seniors (CHAMPS) questionnaire, physical function scale of the SF-36 and physical function (Short Physical Performance Battery (SPPB) test) using Spearman’s correlation coefficients. The test-retest reliability was analyzed using the intraclass correlation coefficient (ICC).

**Results:**

In total, 109 patients completed all of the questionnaires, took part in physical performance tests and had valid data. Their mean age was 64 ± 11 years, with a dialysis history of 31 ± 10 months. For total calories, there was a strong correlation between the Persian version of the LoPAQ and CHAMPS-measured physical activity (rho = 0.85, *p* < 0.001). In addition, the higher physical activity level reported by Persian version of the LoPAQ was also correlated with better self-reported physical function (rho = 0.7, *p* < 0.001) and better physical performance (rho = 0.67, *p* < 0.001). The ICC ranged from 0.65 to 0.78, indicating strong reliability.

**Conclusion:**

The assessment of the validity and reliability of the Persian version of the questionnaire confirmed its suitability for evaluating the level of physical activity.

**Trial registration:**

ClinicalTrials.gov Identifier: NCT05930964, Registered on 05/07/2023. Registered trial name: Validity and Reliability of Persian Version of Low Physical Activity Questionnaire (LoPAQ).

## Background

End-stage kidney disease patients, especially those undergoing hemodialysis (HD) and peritoneal dialysis, experience a high rate of dysfunction, complications, reduced quality of life, hospitalization, and mortality [1, 2]. The etiological factors of these consequences have a wide range, the most important of which are: cardiovascular disorders (CVD), muscle atrophy and malnutrition. All these factors start or intensify with a sedentary lifestyle [1].

Prospective studies on dialysis patients have shown that reducing the daily activity level increases the risk of death in these patients by 60% [3, 4]. Based on this, proper physical activity helps prevent muscle loss, control associated diseases, improve quality of life and reduce mortality in hemodialysis patients [5, 6]. Guidelines for improving the quality of outcomes in dialysis patients state that increased physical activity and regular exercise should be considered as a cornerstone of treatment approaches for patients undergoing dialysis, especially when the goal is to control risk factors of CVD [7, 8].

Due to its importance, a reliable and feasible method for measurement is needed [9, 10]. There are countless tools for quantifying physical activity. Among other considerations, the choice of tool depends on the activity level of the studied population and the results of interest [11]. A group of tools measures the level of physical activity objectively using a pedometer. This measurement method faces many problems, one of them is the lack of appropriate instructions for the number of measurement days [12–14] and the low adherence of patients to this method [15]. In addition, most of the studies that use objective measurement mainly include western countries with medium to high income [12, 14, 16]. Due to the increasing prevalence of kidney disease, especially in East Asian countries and the Middle East, it is necessary to use tools to measure physical activity that are easily available [16]. In addition, a tool such as a pedometer cannot provide us with information about the type and intensity of the activity performed [11].

A number of different physical activity questionnaires have been used to evaluate patients undergoing hemodialysis treatment. It should be noted that this group is very inactive. However, previous studies have relied on questionnaires that were not specifically designed for this population [17, 18]. Many of these tools, focus on moderate-to-vigorous activities [12, 19–21], as these levels have been most associated with health benefits in healthy populations. As a result of focusing on higher intensity activity, this instrument may not accurately represent physical activity or changes in activity levels among highly inactive groups. Because it covers a wider range of activities than most questionnaires and does not evaluate the activity at the lower end of the spectrum [11]. Previous studies have shown that physical activity, even at low levels, is related to the survival of dialysis patients [3, 22]. However, although scores on common physical activity questionnaires categorize participants according to their general activity level, they do not specify the amount or intensity of physical activity performed and therefore do not correspond well to measures that can be used by clinicians and scores may not be sensitive to change with intervention [11]. There are increasing observations that, especially among sedentary populations, time spent sitting may have adverse effects on outcomes independent of activity participation [23–26].

To address these limitations, the “Low Physical Activity Questionnaire (LoPAQ)” was designed by Jansen et al. [11], which emphasizes very low levels of physical activity, especially walking (representing low-level physical activity), determines the amount of calorie consumption for all leisure time activities and takes sitting time as one of the negative evaluation criteria. These characteristics make this questionnaire suitable for hemodialysis patients according to the physical activity characteristics of hemodialysis patients mentioned above. In addition, this questionnaire enables researchers to estimate whether a hemodialysis patient has reached the activity level recommended in the dialysis patient guidelines on a relatively accurate scale. In addition, the English version of this questionnaire has good validity and has a high correlation with one of the most widely used questionnaires for evaluating physical activity in dialysis patients, Minnesota Leisure Time Activity Questionnaire, and various physical performance indicators [11]. Currently, the LoPAQ in hemodialysis patients is only available in English and Chinese and Japanese [11, 27, 28]. The purpose of this study is to translate and adapt it to Persian language and determine its reliability and validity in hemodialysis patients.

## Method

### Trial design & participants

This study was a cross-sectional study conducted to translate, culturally adapt and evaluate the validity and reliability of the Persian (Farsi) version of the LoPAQ in dialysis patients.

Patients undergoing hemodialysis treatment were recruited from three hemodialysis unit in Iran from May 2023 to June 2023.

The inclusion criteria were as follows: (1) age ≥ 18 years; (2) on hemodialysis for ≥ 3 months; (3) able to walk without assistance (walking device such as cane or walker allowed); and (4) ability to provide informed consent and complete the questionnaires. The exclusion criteria were as follows: (1) diagnosis of mental or cognitive disorders; (2) unstable conditions; and (3) hospitalization in the previous 3 months.

### Trial procedures

After providing written informed consent, eligible patients received a demographic questionnaire. Data were collected on demographic characteristics (age, sex, and time on hemodialysis), primary cause of kidney failure, and comorbidities (atherosclerotic heart disease, congestive heart failure, cerebrovascular accident/transient ischemic attack, peripheral vascular disease, dysrhythmia, and other cardiac diseases, chronic obstructive pulmonary disease, gastrointestinal bleeding, liver disease, cancer, and diabetes). Comorbidities were quantified using Charlson comorbidity index (CCI) established for dialysis patients, which included the underlying cause of kidney failure, as well as 11 comorbidities [29].

Participants were interviewed during dialysis (in a midweek session) to complete the questionnaires. Physical performance was measured on the same day before the dialysis session. After two weeks, the participants were interviewed to complete the Persian version of the LoPAQ again to evaluate test-retest reliability.

### Low physical activity questionnaire (LoPAQ)

The Low Physical Activity Questionnaire (LoPAQ) was initially developed by Johansen et al. [11] to evaluate physical activities at a low level for patients with HD. The questionnaire consists of 11 items that assess various parameters of physical activity within the past 7 days as follows:

#### Walking activities

Participants report the frequency and duration of walking for neighborhood strolls, transportation, fitness, or pleasure, as well as instances of no walking.

#### Light activities

Inquiries about engagement in light activities that increase heart rate slightly, such as gardening, bowling, or light housework, with details on frequency and duration.

#### Moderate activities

Questions on participation in moderate activities that cause a noticeable increase in heart rate and limit the ability to sing, including aerobics or moderate housework.

#### Vigorous activities

Assessment of involvement in vigorous activities that significantly raise heart rate and breathing, such as running or playing basketball.

#### Muscle strengthening exercises

Determination of whether participants performed exercises specifically for muscle strengthening.

#### Flexibility exercises

Evaluation of participation in stretching or flexibility exercises.

#### Sedentary behavior

Measurement of time spent in sedentary activities like sitting, watching television, or using a computer.

#### Napping habits

Inquiry about daytime napping habits, including frequency and duration.

#### Sleep duration

Reporting of average nightly sleep duration over the past week.                                                                Work-Related Questions regarding the physical nature of participants’ work, if applicable, including walking and physical exertion like lifting.

Each item is designed to capture both the intensity and frequency of the activities, providing a comprehensive picture of the patient’s physical activity level. The questionnaire also calculates the kilocalories expended during light, moderate, vigorous, and total physical activities. The scoring system allows for categorization into different levels of physical activity, which can be crucial for managing hemodialysis treatment.

The validity of the English version of the LoPAQ was established by its substantial correlations with the Minnesota Leisure Time Activity Questionnaire (rho = 0.62, *p* < 0.001), the Physical Function score of the 36-item Short Form Health Survey (SF-36; rho = 0.64, *p* < 0.001), and physical performance indexes [11].

### Translation and cultural adaption of the Persian version of the LoPAQ

Once we received permission from the original authors for the linguistic adjustments and validation, the Persian version of the LoPAQ was culturally and linguistically adapted. Following the existing guidelines on translation and matching [30, 31] and World Health Organization recommendations [32], two native Persian translators independently translated the questionnaire from English to Persian, with an emphasis on preserving the content instead of conducting a literal translation. One translator was an exercise physiologist who was aware of the study’s purpose and translation, while the other was an official translator who had no knowledge of the study’s purpose. After the translators completed their work, the two versions were compared, and any contradictions or differences were reviewed by the two translators to produce a single translation of the questionnaire. In the next step, two native Persian-speaking translators who had lived in English-speaking countries for an extended period of time translated the Persian translation into English without knowing the study’s purpose. Next, an expert committee composed of various members, including an epidemiologist, two nephrologists, a general practitioner, two sports medicine specialists, and two academic translators, reviewed and discussed the differences between the English translations and the main questionnaire to verify both the linguistic and conceptual alignment of the LoPAQ.

### Measurements

To measure current physical activity behaviors in dialysis patients, we utilized the Community Healthy Adults Model Program for Seniors (CHAMPS) questionnaire. The CHAMPS questionnaire contains 41 questions, each split into two parts, which evaluate the frequency and duration of various physical and non-physical leisure time activities, ranging from light to intense. Each question inquires whether an individual has engaged in a certain activity over the past four weeks, specifically during an average week. If the participant has taken part in the activity during a typical week, they are then asked to specify the frequency and duration for that week. The activities described span from light activities, like casual walking and water exercises, to moderate ones such as cycling or general conditioning to vigorous ones, like jogging or fast-paced swimming [33].

CHAMPS was chosen due to its previous use in similar populations [34–36] and its ability to measure various activities beyond just exercises. The measure provides two scores, including the frequency of activity per week and the estimated caloric expenditure per week. Each activity listed in the questionnaire was assigned a metabolic equivalent of task (MET) value based on the values reported by Ainsworth and colleagues [37], which is a physiological measure expressing the energy cost of physical activities. By multiplying the estimated duration of each activity by the MET value and summing these across all relevant activities, the duration and intensity of total physical activity reported were estimated.

Self-reported physical function was evaluated by administering the Physical Function (PF) scale of the SF-36 [38], which comprises 10 activities that participants are asked to perform and score from 0 to 100. Higher scores indicate better physical function.

Physical performance was assessed using the Short Physical Performance Battery (SPPB) [39] and its individual components, which comprise gait speed, a timed sit-to-stand test, and balance tests. Performance tests were performed immediately before a midweek dialysis session. Gait speed was measured twice over a 15-foot course, and the fastest time was recorded. Patients were timed while performing five consecutive sit-to-stand movements from a standard chair as quickly as possible without the support of their arms. Additionally, patients were timed while standing with their feet side by side, in a semi-tandem position, and in a tandem position for up to 10 s each. A total SPPB score was computed based on the results of the performance tests, with each component scored from 0 (unable to perform) to 4 (best performance), resulting in a total score ranging from 0 to 12 [39].

### Sample size

In data analysis and validity review, the ratio of sample size to the number of items should be at least five to one, and it is better to consider ten to one [40]. This questionnaire has 11 items and considering possible drop-outs 120 participants were required.

### Statistical analysis

Descriptive statistics, including mean, standard deviation (SD), and percentage, were used to present the demographic information. The normality of each variable was assessed by determining the levels of skewness and kurtosis [41]. The content validity of the questionnaire was measured using the content validity index (CVI). A CVI higher than 0.78 is generally considered to indicate good content validity [42].

To assess convergent validity, correlations among the Persian version of LoPAQ and Persian version of the CHAMPS questionnaire, PF scale of the SF-36 and SPPB test were examined using Spearman’s correlation coefficients. A correlation of above 0.40 was considered acceptable [43].

Bland-Altman analyses were used to determine the level of agreement for total energy expenditure per week, derived from the Persian version of LoPAQ and CHAMPS. The test-retest reliability was determined by calculating the intraclass correlation coefficient (ICC, two-way mixed analysis of variance). An ICC > 0.75 represents excellent test-retest reliability, 0.60–0.74 represents good reliability, 0.40–0.59 represents fair reliability, and < 0.4 indicates poor reliability [44]. The LoPAQ has questions, where ask about participation in exercises, and questions ask about participation in regular activities. These two types of questions cover different topics and are inconsistent in essence. Additionally, the first type of questions has three parts asking about different exercise intensities, including strenuous, moderate, and mild exercise, which are also inconsistent intrinsically. Therefore, the authors did not measure Cronbach’s alpha for the whole questionnaire. All statistical tests were two-tailed, and *p* < 0.05 was considered statistically significant. Statistical analyses were performed using IBM SPSS software 25.

## Results

Overall, 151 patients were assessed for eligibility, of whom 120 were consented and recruited for validity test. Among them, 109 patients who completed all of the questionnaires, took part in physical performance tests and had valid data, were included in the data analysis. 85 participants completed Persian version of the LoPAQ for test-retest reliability.

### Baseline characteristics

Characteristics of the patients are displayed in Table 1. This table also includes the total energy expenditure reported on the LoPAQ and CHAMPS questionnaire, as well as sitting time and physical function of participants.

**Table 1:** Patients’ characteristics

Parameters	Total (*n* = 109)
**Male**	61 (56%)
**Age (year)**	64 ± 11
**Hemodialysis history (months)**	31 ± 10
**Primary kidney disease**	
Diabetes	44 (40%)
Hypertension	29 (27%)
Glomerulonephritis	17 (16%)
Other	19 (17%)
**CCI**	5 ± 2
**Physical Activity (kcal/week)**	
LoPAQ total physical activity	780.5 ± 292
CHAMPS total physical activity	821.5 ± 302.1
**Sedentary (min/week)**	
LoPAQ sitting time	1690.5 ± 525
**Physical Function**	
Self-reported*	62.4 ± 10.8
Gait speed (m/s)	0.9 ± 0.2
Sit-to-stand (s)	2.8 ± 1.1
Balance score	2.9 ± 0.9
Total SPPB score	8.8 ± 2.4

Values are as mean (standard deviation) or n (%)

CCI: Charlson Comorbidity Index, LoPAQ: Low Physical Activity Questionnaire, CHAMPS: Community Healthy Adults Model Program for Seniors, SPPB: Short Physical Performance Battery

*: Physical Function score of the SF-36

### Validity estimate

For content validity, the expert panel made some changes to the original English version of the LoPAQ. They commented that the use of “golfing”, “bowling” and “boating (motor)” as examples of “light activity” was not appropriate for Persian HD patients, as they do not typically engage in those exercises. Therefore, these examples were replaced with “watering the plants” and “walking downstairs.” In the subscale of “moderate activity,” “swimming (the side stroke or breast stroke)” were replaced by “Tai-Chi”. Additionally, “softball” and “downhill skiing” were deleted. In the subscale of “vigorous activity,” “playing soccer” and “cross-country skiing” were removed and replaced with “carrying heavy loads” as per the expert panel’s suggestions. These changes were made to ensure that the Persian version of the LoPAQ was culturally appropriate for Persian HD patients.

The CVI was used to measure the content validity of the Persian version of the LoPAQ, and the expert panel’s modifications improved the CVI score to 0.86, indicating good content validity.

### Convergent validity

Based on the Spearman coefficient analysis, the study found strong correlation between the Persian version of the LoPAQ and CHAMPS-measured physical activity for total calories (rho = 0.85, *p* < 0.001). The total physical activity level reported by Persian version of the LoPAQ was also correlated with self-reported physical function (rho = 0.7, *p* < 0.0.5) and better physical performance (rho = 0.67, *p* < 0.05). Additionally, there was a negative correlation between LoPAQ-measured sedentary time and each component of SPPB and self-reported function (Table 2). These findings indicate that the Persian version of the LoPAQ has good criterion validity as it correlates well with the other physical activity level measurements.

**Table 2:** Correlation between LoPAQ measurements and physical function

	LoPAQ total physical activity (kcal/week)		LoPAQ sitting time (min/week)	
Variables	Rho	*p*-value	Rho	*p*-value
**PF scale**	0.7	0.003**	− 0.55	0.02*
**Gait speed, m/s**	0.65	0.02*	− 0.6	0.007**
**Sit-to-stand, s**	-0.72	0.002**	0.65	0.005**
**Balance, s**	0.63	0.03*	− 0.54	0.02*
**SPPB**	0.67	0.02*	− 0.7	0.002**

LOPAQ: Low Physical Activity Questionnaire, PF scale: Physical Function scale of the SF-36, SPPB: Short Physical Performance Battery

*p < 0.05 significant

**p < 0.01 highly significant

### Bland-Altman analysis

**Fig. 1:**
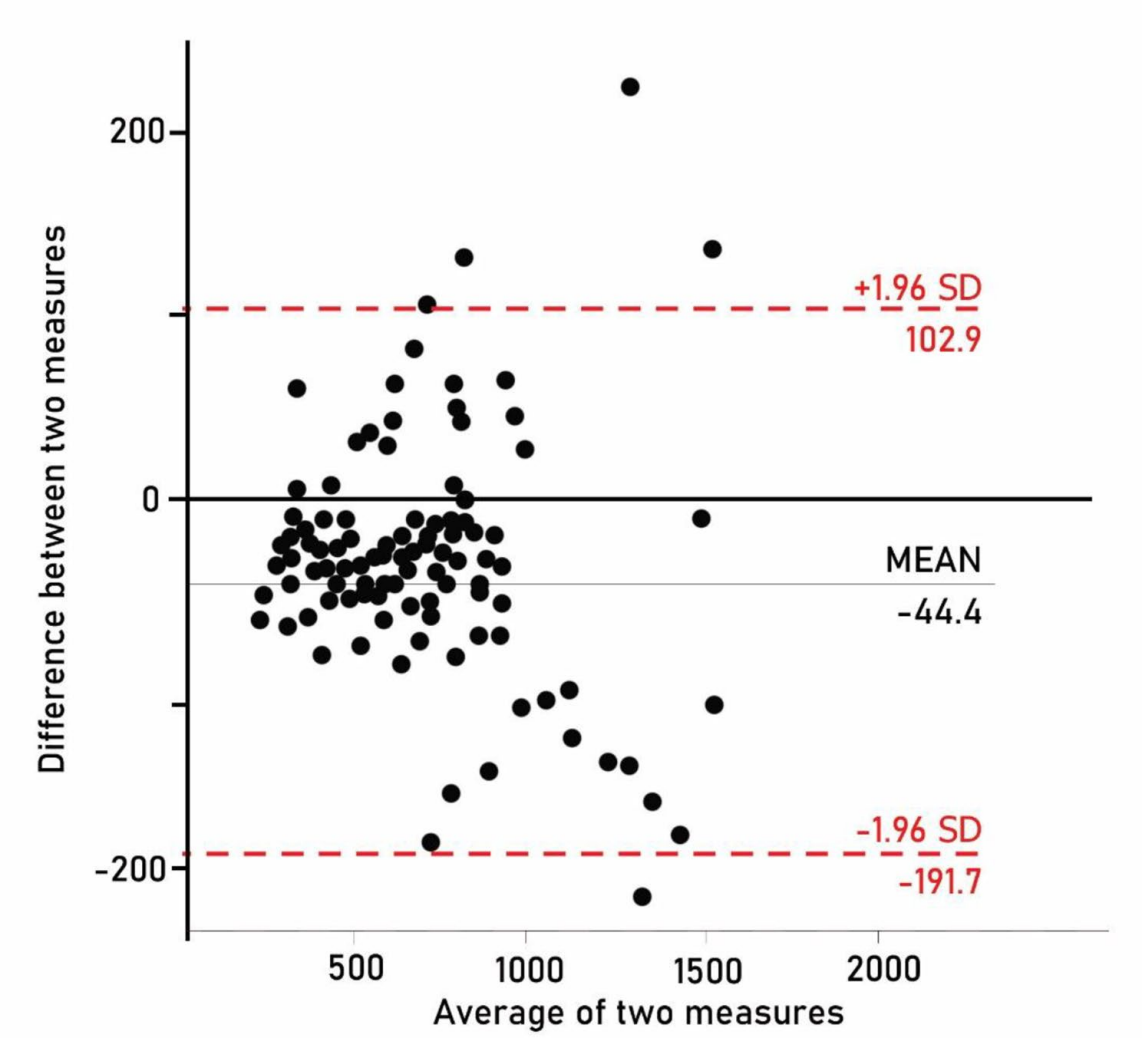
The Blant-Altman plot for total energy expenditure using the LoPAQ and CHAMPS questionnaire

The Bland-Altman plot was used to assess the agreement between the Persian version of the LoPAQ and CHAMPS measurements (Fig. 1). The results showed a mean difference of 44.4 kcal of energy expenditure per week between the two methods. The limits of agreement for total calories between the Persian version of the LoPAQ and CHAMPS had narrow ranges, from − 191.7 to 102.9, with 4 outliers (3.7%). These findings suggest that there is consistency between Persian version of the LoPAQ and CHAMPS measurements.

### Reliability

The mean total energy expenditure of the Persian version of the LoPAQ was 780.5 ± 292 at the first test and 850 ± 310.5 kcal/week at retest.

As stated in the given text, due to the inconsistencies between questions of the LoPAQ, the authors did not measure Cronbach’s alpha for the whole questionnaire. Instead, they reported the intra-class correlation coefficient (ICC) for each question to assess the test-retest reliability of the questionnaire. This approach is appropriate when the questionnaire has multiple subscales or when the questions are not highly correlated with each other, as in the case of the LoPAQ. The ICC assesses the consistency of responses across time for each individual question, rather than measuring the internal consistency of the whole questionnaire. The ICCs ranged from 0.65 to 0.78 for the subscale scores, indicating good to excellent test-retest reliability. The detailed results are shown in Table 3.

**Table 3:** Reliability of the Persian version of the LoPAQ subscales

Subscales	ICC
Light activity energy expenditure (kcal/week)	0.65
Moderate activity energy expenditure (kcal/week)	0.7
Vigorous activity energy expenditure (kcal/week)	0.73
Total energy expenditure (kcal/week)	0.78
Walking time (min/week)	0.65
Sitting time (min/week)	0.68

LoPAQ: Low Physical Activity Questionnaire, ICC: Intra-class correlation coefficient

## Discussion

The physical activity level of patients undergoing HD treatment is low and significantly lower than the same inactive control groups [14, 45]. Research has shown that the level of physical activity of HD patients gradually decreases by approximately 4.5% per month during the entire period of the disease [20]. In addition, Li et al. [21] found that the activities related to the work done at home constitute the largest part of the energy consumption of hemodialysis patients, which means that the types of activities of hemodialysis patients are usually limited to the lower category [12]. Inactivity sets off a vicious cycle in which energy imbalances can increase comorbidities such as hypertension, diabetes, coronary artery disease, depressive disorders, hospitalization rates, and disability. Each of these conditions aggravates the decrease in the quality of life and increases the mortality of patients [15, 14].

Despite numerous studies casing the positive impacts of physical activity on health, there is a notable lack of well-defined, evidence-based exercise programs for patients with chronic kidney disease [46]. For a better understanding of the benefits of physical activity in these patient groups, it’s crucial to have valid methods to assess their physical activity levels. Such measurement tools can potentially encourage these patients to be more active and enhance their physical activity levels [47]. Recall questionnaires are the most frequently used measure for assessing physical activity because of their simplicity, cost-effectiveness, and ease of administration compared to more objective measures [48, 49]. A newly valid assessment tool, LoPAQ has been designed to capture the low activity level among typically sedentary HD patients [11], which includes a 1-week assessment period.

The aim of this study was to develop the Persian version of LoPAQ and to determine its validity and reliability for measuring low levels of physical activity compared with CHAMPS questionnaire and other tools.

It’s important to bear it in mind that Farsi is the primary language spoken across Iran, and it is taught in schools from the early grades, making the questionnaire easy-to-understand for all Iranian citizens. Accordingly, the findings from this questionnaire could be considered applicable throughout various regions of Iran [50]. To ensure that the translated version of the document was consistent with the original, the focused was put on four key areas of equivalence: semantic, idiomatic, experiential, and conceptual [51]. The translation and cultural adaptation process was thoroughly based upon the four-stage framework suggested by Herdman et al. [52].

The main findings indicated that the total energy expenditure reported by the Persian version of LoPAQ also correlated well with the kilocalories obtained from the CHAMPS questionnaire.

Theses finding is in line with the results presented by the initial study for characterization of the LoPAQ, which also used a subjective tool for criterion validity [11].

Furthermore, energy expenditure from the LoPAQ was associated with patients’ self-reported physical functioning and with several tests of physical performance. Sedentary behavior was associated with worse self-reported function and worse performance on the SPPB.

These results were consistence with previous studies with similar comparisons [11, 28], indicating the generalizability of our findings.

The Persian version of the LoPAQ has shown exceptional test-retest reliability, outperforming its Chinese counterpart across all subscales [27]. This discrepancy may be attributed to cultural differences in daily activities and walking behaviors, which are inherently influenced by the distinct social norms and environmental contexts of each region. The original and Japanese versions have not reported on test-retest reliability [11, 28], highlighting a potential area for further research. Additionally, the timing of the studies could contribute to the variability in results; the present study was conducted in Spring, whereas the Chinese study was carried out in Winter. Seasonal weather conditions, including snow, rain, and wind, can introduce significant variability in walking behavior over a two-week period. Therefore, the reliability of the Chinese version of the LoPAQ may have been affected not only by true variability in walking activities but also by potential measurement errors over time.

Despite the LoPAQ demonstrating comparable performance to other validated physical activity questionnaires, it presents certain advantages. Primarily, the LoPAQ places emphasis on walking, making it more responsive to changes in routine physical activity, especially when implementing interventions to promote walking. Additionally, it is tailored to capture activities that are typically part of dialysis patients’ lifestyles and includes the assessment of sedentary behaviors. Lastly, the LoPAQ offers the benefit of brevity compared to widely used questionnaires such as CHAMPS, requiring only 10 min for administration, including instructions provided to participant.

The development of the Persian version of the LoPAQ represents a significant step forward in the assessment of physical activity among HD patients. Its design to capture low levels of activity, which is typical of this patient group, makes it a potentially powerful tool in clinical practice. By integrating the LoPAQ into routine assessments, clinicians can monitor patient activity levels more effectively and identify those who may benefit from interventions aimed at increasing physical activity. Moreover, the LoPAQ’s sensitivity to changes in activity levels makes it an ideal instrument for evaluating the effectiveness of such interventions, thereby contributing to the optimization of patient care and potentially improving health outcomes.

Our study has some limitations that should be addressed. This study focused solely on patients undergoing hemodialysis and did not include an examination of the screening ability of the LoPAQ in patients on peritoneal dialysis (PD). Given the shorter daily dialysis time constraints for PD patients, their lifestyle may differ from that of hemodialysis patients, who typically visit hospitals or clinics three times a week according to their dialysis schedule. Therefore, it is important to further investigate the LoPAQ’s screening ability in dialysis patients, including those on PD. Additionally, this study did not assess the extent to which the LoPAQ is responsive to changes in physical activity. Moreover, there is a limitation in our analysis concerning the impact of dialysis duration on physical activity levels. The literature suggests a negative correlation between extended dialysis treatment and physical activity [53–55], which our study was not equipped to explore due to the limited number of participants stratified by dialysis duration.

Despite this, our findings contribute to the body of knowledge and echo the need for a comprehensive assessment of physical activity in patients undergoing dialysis. We propose that future research should focus on larger cohorts that allow for stratification by dialysis duration to determine if the reliability of the LoPAQ is affected by the length of dialysis treatment.

Understanding this relationship has significant implications for clinical practice, as it may guide healthcare professionals in tailoring interventions to enhance the physical activity and overall well-being of patients on long-term dialysis.

Although the spoken Persian language may vary across regions and countries, the written language remains largely consistent. However, it is essential to consider the potential cultural and socioeconomic variations among Persian-speaking countries that might influence the generalizability of the findings. Cultural sensitivity is crucial in healthcare, as the cultural background of HD patients can significantly affect their treatment compliance and interaction with healthcare services [56]. Moreover, socioeconomic factors such as education level, income, and living conditions have been associated with health disparities and could impact the management and outcomes of HD patients [57]. Therefore, while the linguistic consistency provides a solid foundation for the study, a comprehensive understanding of the cultural and socioeconomic context is imperative to fully appreciate and apply the research outcomes to the broader Persian-speaking countries.

## Conclusion

The Persian version of the LoPAQ had validity and excellent reliability. In addition, it was easier and less time-consuming than previously validated physical activity questionnaires. The LoPAQ demonstrated a good correlation with physical function among dialysis patients and can be used for assessing their physical activity level as well as predicting their performance.

## Data Availability

The datasets used and/or analyzed during the current study are available from the corresponding author upon reasonable request.

## Abbreviations

LoPAQ: Low Physical Activity Questionnaire
HD: Hemodialysis
CHAMPS: Community Healthy Adults Model Program for Seniors
CVD: Cardiovascular Disease
CCI: Charlson Comorbidity Index
MET: Metabolic Equivalent of Task
PF: Physical Function
SPPB: Short Physical Performance Battery
SD: Standard Deviation
CVI: Content Validity Index
ICC: Intraclass Correlation Coefficient
PD: Peritoneal Dialysis
GREX: Global Renal Exercise
